# Antifungal Activity of Commercial Essential Oils and Biocides against *Candida Albicans*

**DOI:** 10.3390/pathogens7010015

**Published:** 2018-01-25

**Authors:** Elisa Serra, Lilia Araida Hidalgo-Bastida, Joanna Verran, David Williams, Sladjana Malic

**Affiliations:** 1 School of Healthcare Science, Manchester Metropolitan University, Manchester M1 5GD, UK; elisa.serra@stu.mmu.ac.uk (E.S.); A.Hidalgo@mmu.ac.uk (A.H.-B.); 2 School of Research, Enterprise and Innovation, Manchester Metropolitan University, Manchester M1 5GD, UK; J.verran@mmu.ac.uk; 3 School of Dentistry, Cardiff University, Cardiff CF14 4XY, UK; WilliamsDD@cardiff.ac.uk

**Keywords:** *Candida albicans*, oral candidosis, commercial essential oils, biocides, antifungal activity, minimum inhibitory concentration, minimal biofilm eradication concentration, cytotoxicity

## Abstract

Management of oral candidosis, most frequently caused by *Candida albicans*, is limited due to the relatively low number of antifungal drugs and the emergence of antifungal tolerance. In this study, the antifungal activity of a range of commercial essential oils, two terpenes, chlorhexidine and triclosan was evaluated against *C. albicans* in planktonic and biofilm form. In addition, cytotoxicity of the most promising compounds was assessed using murine fibroblasts and expressed as half maximal inhibitory concentrations (IC50). Antifungal activity was determined using a broth microdilution assay. The minimum inhibitory concentration (MIC) was established against planktonic cells cultured in a range of concentrations of the test agents. The minimal biofilm eradication concentration (MBEC) was determined by measuring re-growth of cells after pre-formed biofilm was treated for 24 h with the test agents. All tested commercial essential oils demonstrated anticandidal activity (MICs from 0.06% (*v*/*v*) to 0.4% (*v*/*v*)) against planktonic cultures, with a noticeable increase in resistance exhibited by biofilms (MBECs > 1.5% (*v*/*v*)). The IC50s of the commercial essential oils were lower than the MICs, while a one hour application of chlorhexidine was not cytotoxic at concentrations lower than the MIC. In conclusion, the tested commercial essential oils exhibit potential as therapeutic agents against *C. albicans*, although host cell cytotoxicity is a consideration when developing these new treatments.

## 1. Introduction

*Candida* are commensal fungal microorganisms that can colonise the oral cavity, where they are mainly found on the posterior part of the tongue and the oral mucosa. Changes in the oral environment that lead to increased *Candida* growth can instigate oral candidosis [1]. The rising number of immunocompromised and immunodeficient patients has resulted in an increased incidence of fungal infections. To highlight this, *Candida*-related infections affect 65% of HIV positive individuals and over 80% of AIDS patients [2,3,4]. The higher life expectancy of the general population has also led to a rise in denture wearing, with a concomitant increase in *Candida*-associated stomatitis [5,6,7]. Even though more than 17 *Candida* species can cause human infection, oral candidosis are mainly caused by *C. albicans* [8]. In the mouth, *Candida* typically grows as biofilms, which are three-dimensional structures attached to surfaces including human tissue or abiotic substrates (e.g., a denture). Biofilm cells are embedded in a self-produced extracellular polymeric matrix and importantly often exhibit an elevated tolerance to antimicrobial agents and host defences [5].

Current therapies for oral candidosis include use of topical or systemic antifungal agents, such as polyenes and azoles. Polyenes (e.g., nystatin and amphotericin B) are fungicidal through binding to ergosterol in the fungal cell membrane and inducing cell membrane damage. Azoles, such as fluconazole and miconazole, are fungistatic by inhibiting the enzyme lanosterol demethylase, involved in ergosterol biosynthesis [9]. Importantly, the range of available antifungals are limited compared to antibiotics [9] and coupled with the rise of *Candida* resistance, especially within biofilms, this has led to an interest in the discovery of new antifungal compounds [10].

Essential oils are natural products produced by aromatic plants and are mainly composed by terpenes and terpenoids [11]. Being lipophilic, these oils typically integrate into membrane structures causing increased cell permeability, leaching of intracellular components and inactivation of enzymes [12,13]. Essential oils can act against *Candida* by inhibiting ergosterol synthesis [14,15,16,17,18], altering cell wall morphology [15,17,18,19], inhibiting enzymes involved in cell wall synthesis [18,20], changing cell membrane permeability [21,22] and producing oxygen reactive species [23]. Furthermore, essential oils can also interact with the mitochondrial membrane leading to cidal effects [11]. Antimicrobial, anti-aseptic, anti-inflammation and anti-oxidant activity of essential oils, alone and in combination with commercial agents is well known [13,24,25,26]. However, limited knowledge exists regarding essential oil activity against biofilms and also host cell cytotoxicity.

The aim of this study was therefore to investigate the antifungal potential of twelve commercial essential oils and two terpenes (E-cinnamaldehyde and linalool) against *C. albicans* planktonic and biofilm growth. The cytotoxicity of the most active commercial essential oils was established against mouse fibroblasts. Antifungal activity of commercial essential oils was compared to chlorhexidine (CHX) and triclosan. These two biocides have previously shown antimicrobial properties against a wide range of oral pathogens and are frequent components in mouthwashes and toothpastes [27,28].

## 2. Results

### 2.1. Minimum Inhibitory Concentration (MIC) 80 and Minimal Lethal Concentration

The minimum inhibitory concentration (MIC) 80 of the test agents against *C. albicans* NCYC 1363 and *C. albicans* 135BM2/94 are shown in Table 1. The commercial essential oils that inhibited the growth at the lowest concentrations were melissa and geraniol, while myrtle and sage had the lowest fungistatic potential (*p* < 0.001).

Fungicidal activity was also expressed as the lowest concentration of antimicrobial agent that killed the microorganism (minimal lethal concentration) (Table 2). All tested compounds, with exception of triclosan, had minimal lethal concentrations against *C. albicans* at tested concentrations. However, these lethal concentrations were generally higher than the previously established MICs.

### 2.2. Minimal Biofilm Eradication Concentration 80

The antifungal activity of biocides and commercial essential oils against *C. albicans* biofilms was expressed as the minimal biofilm eradication concentration (MBEC) [29]. Most test agents were not active against biofilms at tested concentrations and did not prevent regrowth after removal of the antimicrobial (Table 3). The antimicrobials that exhibited an MBEC against both tested *C. albicans* strains were melissa geranium, E-cinnamaldehyde and linalool (Table 3).

### 2.3. Half Maximal Inhibitory Concentration (IC50) against Fibroblasts

**Figure 1:**
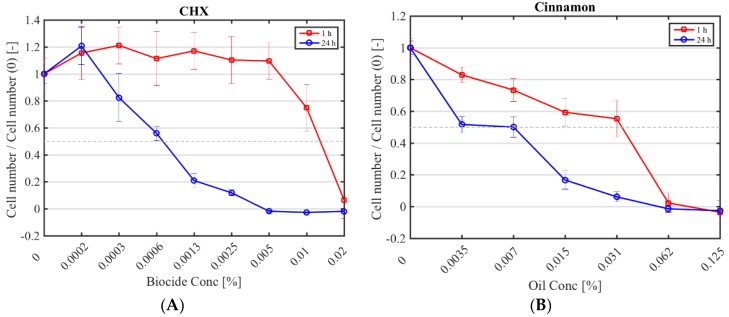

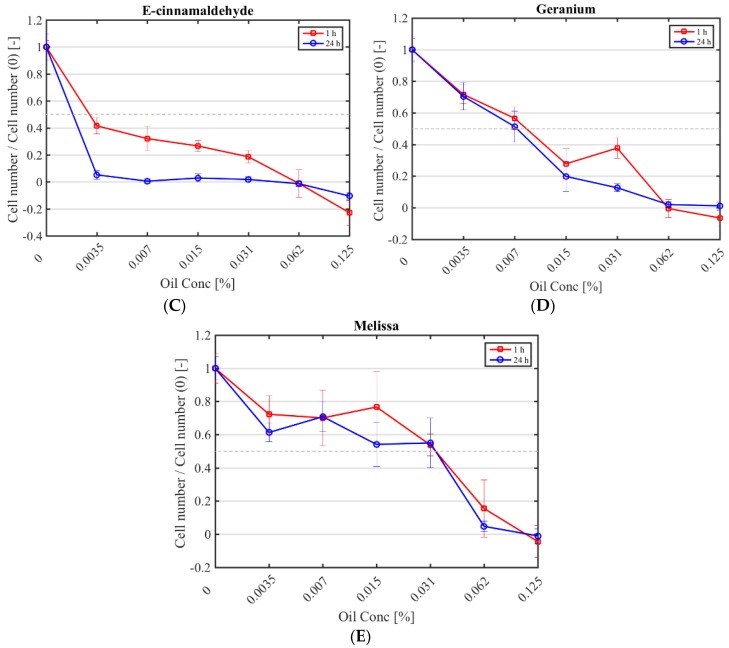
Cytotoxicity of selected antimicrobials against murine fibroblasts. Fibroblast numbers (normalised by the control (0% (*v*/*v*) antimicrobial) after a 1 h (red square) and 24 h application (blue circle) of CHX (**A**); cinnamon (**B**); E-cinnamaldehyde (**C**); geranium (**D**) and melissa (**E**).

The half maximal inhibitory concentration (IC50) CHX, cinnamon, E-cinnamaldehyde, geranium and melissa on fibroblast proliferation after a 1 h and 24 h exposure was determined (Figure 1; Table 4). The highest cytotoxicity occurred with E-cinnamaldehyde, followed by geranium (*p* < 0.0001), which halved proliferation even at the lowest concentration tested. Indeed, a concentration of 0.003% (*v*/*v*) E-cinnamaldehyde and 0.01% (*v*/*v*) geranium inhibited 50% of cell proliferation (Table 4). Melissa was the least cytotoxic commercial essential oil, halving proliferation at 0.03% (*v*/*v*) (*p* < 0.0001). A 1 h exposure of fibroblasts to cinnamon resulted in similar cytotoxicity as melissa but prolonged exposure led to higher cytotoxicity (*p* < 0.0001). A 1 h application of CHX was cytotoxic only at the highest concentration tested (IC50 of 0.01% (*v*/*v*)) which was higher than the MIC, while a 24 h exposure at 7 × 10^−4^% (*v*/*v*) was sufficient to halve fibroblast proliferation.

## 3. Discussion

Essential oils are natural products often extracted from plants and they frequently exhibit antimicrobial, anti-aseptic, anti-inflammatory and anti-oxidant activities. The primary aim of this research was to evaluate the antifungal activity of 12 commercial essential oils against *C. albicans.* All tested commercial essential oils demonstrated antifungal activity against planktonic *C. albicans*, with MICs ranging from 0.06% (*v*/*v*) to 0.4% (*v*/*v*) and MLCs from 0.1% (*v*/*v*) to 1% (*v*/*v*). Comparison of results with those of other studies is problematic given differences in assay techniques [30,31]. In addition, the botanical source, climate and environmental conditions, time of harvesting and extraction method can affect both composition and antimicrobial activity of commercial essential oils [31,32,33].

The effect of plant origin on antimicrobial properties can be appreciated by comparing the activity of cinnamon oil extracted from *Cinnamomum zeylanicum* leaves and *Cinnamomum aromaticum* leaves. Both types of cinnamon oils are from the evergreen cinnamomum plant but *Cinnamomum aromaticum* extract contains a higher amount of E-cinnamaldehyde, which could explain the higher antifungal activity (MICs 0.0006% (*v*/*v*)–0.0096% (*v*/*v*)) [32] compared to the present study using *Cinnamomum zeylanicum* (MIC 0.1% (*v*/*v*)) extract. The impact that the amount of E-cinnamaldehyde has on antifungal properties of an essential oil was also evident in this study (MICs of 0.03% (*v*/*v*) and 0.01% (*v*/*v*)). Geranium and melissa oils exhibited highest antifungal potential. Both commercial oils contain geraniol and citronellol, which are antifungal [34] and likely responsible for the similar antifungal activity of these oils (*p* > 0.90). However, the MIC of melissa oil was lower than that previously reported [35,36]. This present study revealed antifungal effects for bergamot oil (MIC of 0.3% (*v*/*v*) and MLC of 0.5% (*v*/*v*)) which has previously only had limited attention. The MIC of basil oil 0.1% (*v*/*v*) (0.9 g/L) was lower than previously reported, namely 0.5% (*v*/*v*) [30] and 0.312% (*v*/*v*) [32] but comparable to the MIC (1250 μg/mL) found against a fluconazole resistant *C. albicans* strain [15]. The main compound of basil and lavender oils is linalool, which previously has had MICs ranging from 0.06% (*v*/*v*) to 0.12% (*v*/*v*) [37]. Comparing activity of pure linalool to those of basil and lavender oils, the anticandidal activity of terpene was not significantly higher than that of basil (*p* > 0.99). Tea tree oil had an MIC of 0.2% (*v*/*v*) and this was similar to that recorded by Hammer et al. against *C. albicans* [38]. Sage oil exhibited MICs of 0.3% (*v*/*v*) (2.7 g/L) and 0.4% (*v*/*v*) (3.7 g/L), which were comparable to the MIC of 2.78 g/L reported using a disk diffusion method [39] but lower than the MIC of 1.32 mg/mL measured by broth microdilution assay [40]. Despite their differences in composition, peppermint and spearmint oils had similar antifungal activities with MICs of 0.1% (*v*/*v*) and 0.1% (*v*/*v*)–0.2% (*v*/*v*), respectively (*p* > 0.07). However, while the MICs of spearmint oil were similar to those reported by Hammer et al. [30], the MIC of peppermint oil was higher than that found by Those et al. [41]. Myrtle oil had the lowest antifungal potential, even though its MICs were lower than those previously reported by Mahboubi et al. (MIC of 0.8–1.6% (*v*/*v*)) [42]. CHX and triclosan, two biocides whose antimicrobial properties are widely recognised and both commonly added to mouthwashes and toothpastes, were also evaluated in this study. Triclosan exhibited fungistatic activity only at concentrations higher than those used in toothpaste formulations (0.3% (*w*/*v*) [43]) but did not exhibit fungicidal effects at tested concentrations.

The majority of agents had limited antibiofilm activity. Bacteria in biofilms can be between 10 and 1000 times more tolerant to antibiotics than their planktonic counterparts and similar findings have been reported for *Candida* [44]. The mechanisms by which biofilm cells have elevated antimicrobial tolerance are complex and likely multifactorial. These include altered gene expression following surface attachment, reduced growth rates in biofilms, variable nutrient availability that induces changes in phenotype and the presence of extracellular polymeric substances that impedes penetration of agents into the biofilm [45]. Few studies have previously reported activity of commercial essential oils or biocides against *C. albicans* biofilms [46,47]. In the present study, from melissa oil, geranium oil, E-cinnamaldehyde and linalool all had anti-biofilm activity, whilst CHX only had anti-biofilm activity against *C. albicans* NCYC 1363. A 3 min application of cinnamon (1 mg/mL) and citronella (1 mg/mL) oils has been found to reduce biofilm cell numbers immediately after treatment but this effect was not evident 48 h post treatment [46]. These results concur with the current study, where no antibiofilm activity was noted for cinnamon and citronella oils after 24 h. An MBEC of tea tree oil of 12.5% (*v*/*v*) had previously been reported [47], which is a higher concentration (8% (*v*/*v*)) than tested in this study, as difficulties were encountered in forming a stable suspension of the oil-medium using 1% (*v*/*v*) Tween 80.

Few studies have investigated the cytotoxic effects of these oils. Cytotoxicity of CHX, cinnamon, E-cinnamaldehyde, geranium and melissa oils had a dose- and time-dependent cytotoxicity. Overall, the commercial essential oils halved fibroblast proliferation at concentrations lower than their MICs. The IC50 values for E-cinnamaldehyde, geranium and cinnamon oils were actually 10-fold lower than their MIC 80, while melissa oil had an MIC 80 of 0.06% (*v*/*v*) and an IC50 of 0.03% (*v*/*v*). Although a different assay and cell type was used, the melissa oil results (IC50 0.3 g/L) were in accordance with those of Paul et al. [48] who did not see a significant change in leukocytes viability after 3 h treatment with 150 μg/mL melissa oil. Several studies have used E-cinnamaldehyde to inhibit proliferation of cancer cells and reported IC50s ranging from 45.8 to 129.4 mM [49], higher than those obtained in this study with fibroblasts (0.16–0.26 mM). Barros et al. found that at concentrations lower than those evaluated in this study (5 µg/mL), *Cinnamomum zeylanicum* oil had cytoxicity towards erythrocytes [50]. A 1 h exposure of fibroblasts to CHX (0.01% (*v*/*v*)) halved cell proliferation compared to controls. However, this concentration was lower than the MICs (2.5 × 10^−3^% (*v*/*v*) and 5 × 10^−3^% (*v*/*v*)) found in the current study. This finding was similar to the cytotoxic effect of CHX previously reported using macrophages [51] and human alveolar bone cells [52]. Even if these results showed that commercial essential oils were cytotoxic, it should be taken into account that cytotoxicity was conducted in 2D culture, which is notably different from in vivo conditions. Further investigation on mammalian cells could be performed in 3D culture or *ex/in vivo* models to better mimic the biological structure of the tissues.

## 4. Materials and Methods

### 4.1. Essential Oils and Biocides Preparation

Twelve commercial essential oils (Essential Oils Direct Ltd., Oldham, UK) (Table 5), two terpenes (E-cinnamaldehyde and linalool (Sigma-Aldrich, Gillingham, UK)), chlorhexidine digluconate (CHX) (Sigma-Aldrich, Gillingham, UK) and triclosan (Irgasan from Sigma-Aldrich, Gillingham, UK) were evaluated.

The commercial essential oils were tested at a range of concentrations against planktonic growth (2% (*v*/*v*) to 0.007% (*v*/*v*) and biofilms (8% (*v*/*v*) to 0.125% (*v*/*v*)). All agents were prepared in Sabouraud Dextrose Broth (SDB; Oxoid Ltd, Basingstoke, UK). To enhance dispersion of essential oils in the medium, 1% (*v*/*v*) Tween 80 (Sigma-Aldrich, Gillingham, UK) was added. In the case of biofilm studies, 0.015% (*w*/*v*) Agar Bacteriological (LP0011 Oxoid) was added to SDB [53]. CHX was used in SDB at concentrations between 0.04% (*v*/*v*) to 3.1 × 10^−4^% (*v*/*v*) and from 0.08% (*v*/*v*) to 6.2 × 10^−4^% (*v*/*v*) for planktonic and biofilm growth experiments, respectively. A 20% (*w*/*v*) stock solution of triclosan was prepared in Dimethyl Sulfoxide (DMSO) (Fisher Chemical, Loughborough, UK). Serial doubling dilutions of the stock solution were prepared in SDB yielding final concentrations from 5.2 × 10^−6^% (*v*/*v*) to 6.7 × 10^−4^% (*v*/*v*) and from 1.7 × 10^−4^% (*v*/*v*) to 5 × 10^−3^ (*v*/*v*) for planktonic and biofilm experiments, respectively.

### 4.2. Microorganisms

*Candida albicans* NYCY 1363 and *C. albicans* 135BM2/94 were used to assess antifungal activity of commercial essential oils and biocides. *Candida albicans* 135BM2/94 is a clinical strain from the School of Dentistry (Cardiff University), which has been described as a high invader of tissues [54]. Strains were subcultured onto Sabouraud Dextrose Agar (SDA) (CM0041 Oxoid) and grown at 37 °C in an aerobic incubator for 24 h. A colony of *C. albicans* was inoculated in 20 mL of SDB and incubated aerobically with shaking (150 rev/min) overnight at 37 °C. The overnight culture was prepared in SDB to a turbidity equivalent to a 0.5 McFarland Standard and used for further experiments.

### 4.3. Minimum Inhibitory Concentration and Minimal Lethal Concentration

The minimum inhibitory concentration (MIC) and the minimal lethal concentration (MLC) were determined using a broth microdilution assay. The method was adapted from that previously reported by Malic et al. [29]. Briefly, 100 µL of antimicrobial and 100 µL of overnight culture diluted to 1 × 10^5^ CFU/mL were added to the wells of 96-well microtitre plates (Thermo Fisher Scientific, Hemel Hempstead, UK). Controls included *Candida* suspension cultured in SDB, with or without 0.5% (*v*/*v*) of Tween 80. In addition, when triclosan was tested, SDB containing 1% (*v*/*v*) DMSO was used as control. The plates were covered with the lids supplied by the manufacturer and sprayed with 3% (*v*/*v*) of Triton 100-X (Sigma-Aldrich, Gillingham, UK) in pure ethanol to reduce condensation. The plates were incubated aerobically at 37 °C with shaking at 110 rpm, for 24 h. Growth was estimated by measuring turbidity of each well by spectrophotometric absorbance at 620 nm (Thermo Scientific™ Multiskan™ GO Microplate Spectrophotometer), shaking 3 s before the reading. The absorbance readings were standardised against microbial-free controls. The minimal inhibitory concentration 80 (MIC 80) was defined as the lowest concentration of the antimicrobial agent that showed at least 80% reduction in absorbance compared to the control. The MLC was determined by plating selected well contents (where no visible growth was evident) on to SDA and incubating for 24 h at 37 °C. The MLC was defined as the lowest concentration of antimicrobial agent that killed the *Candida* as shown by no colony growth on SDA. All concentrations were tested in quadruplicate and on three separate occasions.

### 4.4. Minimal Biofilm Eradication Concentration 80

The minimal biofilm eradication concentration (MBEC) method was adapted from Malic et al. (2013) [29]. Briefly, a 96-well microtitre plate containing 200 µL of an overnight culture diluted at 1 × 10^5^ CFU/mL was incubated for 48 h at 37 °C without agitation to allow biofilm formation. Controls included *Candida* suspension cultured in SDB, with or without 1% (*v*/*v*) of Tween 80 and 0.015% (*w*/*v*) Agar Bacteriological. When triclosan was tested, SDB containing 8% (*v*/*v*) DMSO was also used as control. After 48 h, the SDB was removed and the microtitre plate inverted onto tissue paper to remove residual medium. The biofilm was washed three times with 100 µL of PBS. One hundred µL of test agent was added to the biofilm and the plate incubated statically for 24 h at 37 °C. After incubation, test agent was removed and the biofilm washed twice with 100 µL of PBS. Two hundred µL of SDB was added to each well and the biofilm disrupted by repeated pipetting. The three replicates were then pipetted into a microcentrifuge tube which was then centrifuged for 3 min at 3000 rev/min (Hettich Universal Mikro 12–24, Hettich, Tuttlingen, Germany). The supernatant containing residual test agent was discarded and the microorganisms resuspended in fresh SDB and three wells of a 96-well plate were inoculated with the suspension. The turbidity of the suspension was measured by spectrophotometer absorbance at 620 nm prior to and after incubation for 24 h at 37 °C with shaking at 110 rev/min. The minimal biofilm eradication concentration 80 (MBEC80) was defined as the lowest antimicrobial concentration that prevented at least 80% regrowth of *Candida*. All experiments were conducted on three separate occasions.

### 4.5. Half Maximal Inhibitory Concentration

Mouse fibroblasts (NIH 3T3; Sigma-Aldrich, Gillingham, UK) were cultured in Dulbecco Modified Eagle Medium (DMEM, Sigma) supplemented with 10% (*v*/*v*) foetal bovine serum (FBS) (Gibco, BRL), 1% (*v*/*v*) penicillin/streptomycin (Sigma-Aldrich, Gillingham, UK) and 1% (*v*/*v*) l-glutamine (Sigma-Aldrich, Gillingham, UK). Serial doubling dilutions of commercial essential oils and biocides were prepared in the fibroblast culture medium at final concentrations ranging from 0.25% to 0.007% (*v*/*v*) for the commercial essential oils and from 0.04% to 3 × 10^−4^% (*v*/*v*) for chlorhexidine. Fibroblasts were harvested using trypsin EDTA (EDTA 0.25% (*w*/*v*), Trypsin 0.53 mM, Thermo Fisher Scientific, Hemel Hempstead, UK) and diluted to a density of 5 × 10^5^ cells/mL. One-hundred µl of the cell suspension was used to inoculate a 96-well plate (5 × 10^4^ cells per well) which was then incubated at 37 °C and 5% CO_2_ for 1.5 h. A 100-µL volume of the antimicrobial was then added. After 1 and 24 h, the medium was removed and the cells washed twice with 100 µL of PBS. Three hundred µL of DMEM containing 10% (*v*/*v*) of alamarBlue (AlamarBlue Cell Viability Reagent, Invitrogen) was added to each well and the plate incubated for 1.5 h. Fluorescence was read with a Synergy HT plate reader (BioTek^®^ Instruments, Winooski, VT, USA) with excitation and emission wavelengths of 545 nm and 590 nm, respectively. The half maximal inhibitory concentration (IC50) was defined as the antimicrobial concentration that inhibited 50% cell proliferation compared to the control (i.e., DMEM without antimicrobial agent). Each condition was studied in triplicate and on three separate occasions.

### 4.6. Statistical Analysis

Statistical analysis was performed using GraphPad Prism Version 7.0 (GraphPad Software, Inc., La Jolla, CA, USA). Data were presented as arithmetic mean ± SD. The difference between treatments was statistically analysed using one-way analysis of variance (ANOVA) followed by Tukey multiple comparisons test. Statistically significant differences were set at *p* < 0.05.

## 5. Conclusions

This study showed that all the twelve commercial essential oils, two terpenes and triclosan and CHX had antifungal activity against planktonic *C. albicans*. Six of these compounds (CHX, cinnamon, E-cinnamaldehyde, linalool, geranium and melissa) were also active against *C. albicans* biofilms, which are usually challenging to effectively inhibit. Cytotoxicity screening revealed that the commercial essential oils halved fibroblast proliferation at concentrations lower than those required to inhibit *C. albicans* growth. Further investigation on the effect of these agents against mammalian cells is however warranted before any in vivo application. The antifungal potential of these essential oils could be a future therapeutic for topical candidosis as an option to overcome emerging antifungal drug resistance.

## Figures and Tables

**Table 1:** Minimum inhibitory concentration 80 of commercial essential oils and biocides against *C. albicans* NYCY 1363 and *C. albicans* 135BM2/94 in the planktonic form.

Antimicrobial	Minimum Inhibitory Concentration 80 [% (*v*/*v*)] [(g/L)]	
	*C. albicans* NYCY 1363	*C. albicans* 135BM2/94
Basil	0.1 (0.9)	0.1 (0.9)
Bergamot	0.3 (2.6)	0.3 (2.6)
Cinnamon	0.1 (1.0)	0.1 (1.0)
Citronella	0.1 (0.9)	0.1 (0.9)
Geranium	0.07 (0.6)	0.06 (0.5)
Lavender	0.2 (1.8)	0.1 (0.9)
Melissa	0.06 (0.5)	0.06 (0.5)
Myrtle	0.4 (3.5)	0.3 (2.7)
Peppermint	0.1 (0.9)	0.1 (0.9)
Sage	0.4 (3.7)	0.3 (2.7)
Spearmint	0.2 (1.6)	0.1 (1.1)
Tea tree oil	0.2 (1.8)	0.2 (1.8)
E-cinnamaldehyde	0.03 (0.3)	0.01 (0.1)
Linalool	0.1 (0.9)	0.1 (0.9)
CHX	2 × 10^−3^ (2.1 × 10^−2^)	5 × 10^−3^ (5.3 × 10^−2^)
Triclosan	5.66 × 10^−4^ (8.4 × 10^−3^)	5.89 × 10^−4^ (8.8 × 10^−3^)

Minimal inhibitory concentration 80 (MIC80) defined as the lowest concentration of the antimicrobial agent that led to 80% reduction in absorbance compared to controls without agent. MIC values are in % (*v*/*v*) and in brackets are the equivalent MIC values in (g/L).

**Table 2:** Minimal lethal concentration of commercial essential oils and biocides against *C. albicans* NYCY 1363 and *C. albicans* 135BM2/94 in the planktonic growth mode.

Antimicrobial	Minimal Lethal Concentration [% (*v*/*v*)] [(g/L)]	
	*C. albicans* NCYC 1363	*C. albicans* 135BM2/94
Basil	0.5 (4.5)	0.5 (4.5)
Bergamot	0.5 (4.4)	0.5 (4.4)
Cinnamon	0.1 (1.0)	0.1 (1.0)
Citronella	0.1 (0.9)	0.1 (2.7)
Geranium	0.1 (0.9)	0.1 (0.9)
Lavender	0.5 (4.4)	0.3 (2.6)
Melissa	0.1 (0.9)	0.1 (0.9)
Myrtle	1 (8.8)	1 (8.8)
Peppermint	0.3 (2.7)	0.1 (0.9)
Sage	1 (9.2)	1 (9.2)
Spearmint	1 (9.2)	1 (9.2)
Tea tree oil	0.5 (4.5)	0.3 (2.7)
E-cinnamaldehyde	0.03 (0.3)	0.03 (0.3)
Linalool	0.3 (2.6)	0.3 (2.6)
CHX	2.5 × 10^−3^ (2.7 × 10^−2^)	5 × 10^−3^ (5.3 × 10^−2^)
Triclosan	NA	NA

Minimal lethal concentration was defined as the lowest concentration of the antimicrobial agent that killed *C. albicans.* MLC values are in % (*v*/*v*) and in brackets are the equivalent MLC values in (g/L). NA = no antimicrobial activity at tested concentrations.

**Table 3:** Minimal biofilm eradication concentration 80 of commercial essential oils and biocides against *C. albicans* NCYC 1363 and *C. albicans* 135BM2/94.

Antimicrobial	Minimal Biofilm Eradication Concentration 80 [% (*v*/*v*)] [(g/L)]	
	*C. albicans* NYCY 1363	*C. albicans* 135BM2/94
Basil	NA	NA
Bergamot	NA	NA
Cinnamon	NA	NA
Citronella	NA	NA
Geranium	2.5 (22.3)	2 (17.9)
Lavender	NA	NA
Melissa	1.5 (13.3)	1.5 (13.3)
Myrtle	NA	NA
Peppermint	NA	NA
Sage	NA	NA
Spearmint	NA	NA
Tea tree oil	NA	NA
E-cinnamaldehyde	0.8 (8.4)	0.8 (8.4)
Linalool	1 (8.7)	1.5 (13.1)
CHX	0.07	NA
Triclosan	>5 × 10^−3^ (7.45 × 10^−2^)	>5 × 10^−3^ (7.45 × 10^−2^)

Minimal biofilm eradication concentration 80 (MBEC80) defined as the lowest antimicrobial concentration that prevented at least 80% regrowth of *Candida*, after the biofilm was treated with antimicrobials for 24 h. MBEC values are in % (*v*/*v*) and in brackets are the equivalent MBEC values in (g/L). NA = no antimicrobial activity at tested concentrations.

**Table 4:** Half maximal inhibitory concentration (IC50) against fibroblasts after 1 h and 24 h application of the antimicrobial.

Antimicrobial	Half Maximal Inhibitory Concentration [% (*v*/*v*)] [(g/L)]	
	1 h	24 h
Cinnamon	0.03 (0.36)	0.01 (0.11)
Geranium	0.01 (0.08)	0.01 (0.07)
Melissa	0.03 (0.3)	0.03 (0.3)
E-cinnamaldehyde	0.003 (0.03)	0.002 (0.02)
CHX	0.01 (0.15)	7.32 × 10^−4^ (0.008)

Half maximal inhibitory concentration (IC50) defined as the antimicrobial concentration that inhibits the 50% of cell proliferation compared to controls without agent. IC50 values are in % (*v*/*v*) and in brackets are the equivalent IC50 values in (g/L).

**Table 5:** List of commercial essential oils tested.

Plant Species	Essential Oil	Origin
*Ocimum basilicum*	Basil oil	Leaves
*Citrus bergamia*	Bergamot FCF oil	Peel
*Cinnamomum zeylanicum*	Cinnamon leaf oil	Leaves
*Cymbopogon winterianus*	Citronella oil	Aerial parts
*Pelargonium graveolens*	Geranium oil	Flowering herb
*Lavandula angustifolia*	Lavender oil	Flowering herb
*Melissa officinalis*	Melissa oil	Leaves and tops
*Myrtus communis*	Myrtle oil	Leaves
*Mentha piperita*	Peppermint oil	Whole plant
*Salvia officinalis*	Sage oil	Leaves
*Mentha spicata*	Spearmint oil	Aerial parts
*Melaleuca alternifolia*	Tea tree oil	Leaves and twigs
